# Supplementary material for: MetaRibo-Seq measures translation in microbiomes
Source: Nat Commun. 2020 Jun 29;11:3268. doi: 10.1038/s41467-020-17081-z (PMC7324362; doi:10.1038/s41467-020-17081-z)
Supplement: Supplementary file 10 — Supplementary Data 7 [file 41467_2020_17081_MOESM10_ESM.zip › File2/Confidence_High_Taxonomy/354980_out.krona.html]

Javascript must be enabled to view this page.

members
magnitude
magnitudeUnassigned
count
unassigned
taxon
rank

354980\_out

2

2
superkingdom
2

2
phylum
976

2
class
200643

2
order
171549

family
815
1

1
816
genus

1

SRS143342\_contig\_number\_2597
246787
species

family
2005525
1

375288
genus
1

1262913
species

SRS012849\_contig\_number\_contig-100\_1827.168483
1
